# Cooling after successful resuscitation in cardiac surgery patients

**DOI:** 10.1186/1749-8090-8-190

**Published:** 2013-09-23

**Authors:** Marcel Vollroth, Knut Roehrich, Carlos Correia, Joerg Seeburger, Thilo Noack, Philipp Kiefer, Michael Hoebartner, Martin Misfeld, Farhad Bakhtiary, Martin Kostelka, Friedrich Wilhelm Mohr

**Affiliations:** 1 Department of Cardiac Surgery, Heart Center Leipzig University, Struempellstrasse 39, 04289, Leipzig, Germany

**Keywords:** Cardiac arrest, Therapeutic hypothermia, Neurological outcome

## Abstract

**Background:**

Despite many years of intensive research sudden cardiac death is one of the most common causes of death all over the world. The European Resuscitation Council (ERC) recommends the use of moderate therapeutic hypothermia for 12–24 hours to improve neurological outcome. However, the beneficial effect of this therapy on outcomes for cardiac surgery patients with In- Hospital- Resuscitation (IHR) has not been well studied.

The purpose of this single center analysis was to investigate our first experience in a non – selected IHR population, where hypothermia was induced independent from initial heart rhythm disturbance.

**Method:**

A total of 20 resuscitated patients who were treated in our institution between January 2010 and December 2011 formed the study cohort.

**Results:**

In all patients post- resuscitation course was significantly prolonged with severe low cardiac output syndrome in six patients (30%). Overall four patients (20%) sustained septicemia with the need for high dose inotropic support. The 30 day mortality was 30% (six of twenty). However, stroke with severe neurological impairment appeared in only four patients (20%) after resuscitation with subsequent therapeutic hypothermia.

**Conclusion:**

With our observation study we could demonstrate the benefits for neurological outcome due to therapeutic hypothermia in cardiac surgery patients after successful resuscitation. However post- resuscitation treatment should focus on sufficient therapeutic strategies to avoid the distinctive short term morbidity and mortality.

## Background

Despite many years of intensive research, sudden cardiac death is one of the most common causes of death in the United States and Europe [1]. The European Resuscitation Council (ERC) and the International Liaison Committee on Resuscitation (ILCOR) recommend the use of moderate therapeutic hypothermia for 12–24 hours to improve neurological outcome in patients who survive Out- Of- Hospital Resuscitation (OHR) due to ventricular fibrillation. Based on two randomized trials and one meta-analysis, survivors of OHR have been treated with hypothermia in several European centers since 2003 [2,3]. However, the beneficial effect of this therapy for other heart rhythm disorders and for patients who survived In- Hospital Resuscitation (IHR) has not been well studied. In 2005 the ERC issued the edition of guidelines for resuscitation included an explicit section on the resuscitation concept for cardiac surgery patients after cardiac surgery [4]. This paper encouraged many clinicians in cardiac surgery centers to evaluate their patients more carefully for therapeutic hypothermia after resuscitation.

The purpose of our single center analysis was to investigate our first experience in a non – selected post cardiac surgery patient population, where hypothermia was induced independent from initial heart rhythm disturbance.

## Methods

A total of 20 resuscitated patients who were treated in our institution between January 2010 and December 2011 formed the study cohort. The study protocol (observational study) was approved by our clinical committee. The following inclusion criteria were defined:

1) All heart rhythm disorders (ventricular fibrillation, pulse less electrical activity, and asystole)

2) Age: 18–80 years

3) An interval of maximum 60 min from collapse to return of spontaneous circulation

4) Stable hemodynamic parameters with moderate inotropic support

Patients were excluded if they met any of the following criteria:

1) Response of verbal commandos after successful resuscitation

2) Age: ≤18 and ≥ 80 years

3) Pregnancy

4) An interval of more than 60 minutes of resuscitation

5) Major bleeding

6) Highly inotropic support

7) Terminal illness that preceded the arrest

### Patients

The mean age of the hypothermia treated patients was 70 ± 11.6 years and 4 patients were female (20%). Initial performed surgical procedures were coronary- arterial- bypass grafting (CABG) in 13 patients (65%), aortic valve replacement in 7 patients (35%) and mitral valve replacement in five patients (25%). The average operating time was 219.9 ± 84.7 min. The mean duration of cardiopulmonary bypass was 97.3 ± 85.5 min. All important preoperative patient characteristics are shown in Table 1. Table 2 illustrates indications for cardiac surgery and perioperative data.

**Table 1:** Preoperative characteristics of all 20 hypothermia treated patients

**Demographics**	**% (n)**
**Age**	70 ± 11.6
**Sex**/**male**	80% (16)
**Reason for CPR**: **ASY**/**VF**	40 % (8)/60% (12)
**BMI**	29 ± 3.2
**NYHA**	3.1 ± 0.6
**EF**	48.8 ± 17
**Preoperative MI**	40% (8)
**Diabetes**	30% (6)
**PAVK**	25% (5)
**Creatinine mg**/**dl**	1.7 ± 1.4
**History of stroke**	5% (1)
**COPD**	10% (2)
**Dialysis**	10% (2)
**Emergency surgery**	15% (3)

*ASY* Asystole, *VF* Ventricular fibrillation, *NYHA* New York Heart Association Classification, *BMI* Body-Mass-Index, *EF* Ejection fraction, *MI* Myocardial infarction, *PAVK* Peripheral vascular disease, *COPD* Chronic obstructive pulmonary disease.

**Table 2:** Indication for cardiac surgery and perioperative data of all 20 patients undergoing hypothermia after IHR

**Surgical procedure**	**% (n)**
**CABG**	65% (13)
**AV Replacement**	35% (7)
**MV Replacement**	25% (5)
**MV Repair**	15% (3)
**TV Repair**	10% (2)
**Aortic surgery**	10% (2)
**AF Ablation**	10% (2)
**ASD Closure**	5% (1)
**Duration of surgery**	219.9 ± 84.7 min
**Duration of CPB**	97.3 ± 85.5 min
**Aortic clamp time**	60.4 ± 49.4 min

*CABG* Coronary artery bypass graft surgery, *AV* Aortic valve, *MV* Mitral valve, *AF* Atrial fibrillation, *ASD* Atrial septal defect, *CPB* Cardio pulmonary bypass.

In accordance to the above defined criteria in 8 patients (40%) asystole was the reason for resuscitation and subsequent hypothermia. Ventricular fibrillation causes cardiac arrest in 12 patients (60%). In all patients hypothermia was applied as soon as feasible by intravascular cooling with the COOLGARD 3000 Thermal Regulation System (Zoll Intravascular temperature management, Chelmsford, MA, USA). The patients were cooled down to a target body core temperature of 32–33°C.

Our goal was to reach the target temperature within 2 hours after successful resuscitation. Therapeutic hypothermia was maintained for 24 hours and the patients were then re- warmed with a rate of 0.35°C/ h to a target temperature of 37°C. After re- warming sedation was stopped to evaluate neurological status.

Intensive care treatment was in relation to international ICU standards. All patients were deeply sedated by intravenous administration of methohexital or midazolam and sufentanyl. When clinically appropriate, the patients received neuromuscular blocker intravenously using pancoronium.

### Statistical analysis

Follow-up was 100% completed. Data included patient’s demographics, indication for surgery and early outcome (30 days). The resuscitation and hypothermia data were registered in standardized protocols. Continuous variables are expressed as means ± standard deviations throughout the manuscript.

## Results

Between 2010 and 2011 a total of 20 patients underwent therapeutic hypothermia after IHR. In twelve patients (60%) ventricular fibrillation caused cardiac arrest with subsequent resuscitation, whereas asystole was causal in eight patients (40%). In all patients post- resuscitation course was significantly prolonged and included low cardiac output syndrome in six patients (30%). Acute renal failure was observed in nine patients (45%). Respiratory failure with the need for tracheotomy occurred in 10 patients (50%). Stroke with severe neurological impairment appeared in only four patients (20%) after therapeutic hypothermia. Overall four patients (20%) sustained septicemia with the need for high inotropic support. In five patients (25%) gastro- intestinal bleeding prolonged the post- resuscitation progress.

The 30 day mortality was 30% (six of twenty). In all deceased patients low cardiac output syndrome was causal for death. The following Table 3. presents the postoperative course and short-term mortality.

**Table 3:** **Postoperative course and short**-**term mortality**

**Early outcome**	**% (n)**
**30 day mortality**	**30% (6)**
**Low cardiac output**	30% (6)
**Postop myocardial infarction**	15% (3)
**Cerebrovascular event**	**20% (4)**
**Acute renal failure**	45% (9)
**Sepsis**	20% (4)
**Gastrointestinal Bleeding**	25% (5)
**Tracheotomy**	50% (10)
**Pacemaker implantation**	5% (1)

## Discussion

We herein report our first single center results of in- hospital resuscitated patients treated with therapeutic hypothermia after previous cardiac surgery. Due to post- resuscitation brain damage as the leading cause of death, the prognosis and patients neurological outcome remains often poor. Two prospective randomized trials and various retrospective analysis supported that therapeutic hypothermia after ventricular fibrillation cardiac arrest improves neurologic outcome [1,2,5]. Nevertheless, this therapeutic benefit has not been finally demonstrated in patients with return of circulation from non- shockable rhythms and in patients after in- hospital resuscitation [6]. In our study we observed patients after previous cardiac surgery resuscitated from cardiac arrest due to shockable and non- shockable arrhythmia who were treated with therapeutic hypothermia to a body core temperature between 32 and 33°C for a period of 24 hours.

Our results demonstrate that IHR in cardiac surgery patients lead to severe peri- and post morbidities. However, in order to previously published data we could demonstrate a significant improvement in neurological outcome [7,8]. Stroke with severe neurological impairment was diagnosed in only four patients after successful resuscitation with subsequent hypothermia. Due to previous cardiac surgery, low- cardiac- output syndrome with subsequent multi organ failure causes death in all deceased patients. With our study we could underline the benefits for neurological outcome due to therapeutic hypothermia independently from initial arrhythmia. However, hemodynamic and respiratory failure seems to be more challenging after IHR in cardiac surgery patients.

## Conclusions

The use of therapeutic hypothermia in resuscitated cardiac- surgery patients is associated with improved neurologic outcome independently from initial heart rhythm disturbance. However, post resuscitation care should focus on sufficient cardio- pulmonary treatment strategies to avoid the distinctive short term mortality.

### Consent

Written consent was obtained from the patients for publication of this report.

## Competing interests

The authors declare that they have no competing interests.

## Authors’ contributions

MV, TN, MH treated the patients on ICU. MV wrote the manuscript. CC, KR, JS, PK, FB, MK helped revise the manuscript. All authors read and approved the final manuscript.

## References

[B1] Jamin C Hamilton SE Implementation of hypothermia after cardiac arrest in the intensive care unit ICU Dir 2011 8 97 10.1177/1944451611417180

[B2] The hypothermia after cardiac arrest study group Mild therapeutic hypothermia to improve the neurological outcome after cardiac arrest N Engl J Med 2002 8 549 556 11856793 10.1056/NEJMoa012689

[B3] Lundbye JB Rai M Ramu B Hosseini- Khalili A Li D Slim HB Bhavnani SP Nair SU Kluger J Therapeutic hypothermia is associated with improved neurologic outcome and survival in cardiac arrest survivors of non- shockable rhythm Resuscitation 2012 8 202 207 10.1016/j.resuscitation.2011.08.005 21864480

[B4] Soar J Deakin CD Nolan JP Abbas G Alfonzo A Handley AJ Lockey D Perkins GD Thies K European RC European Resuscitation Council guidelines for resuscitation 2005. Section 7. Cardiac arrest in special circumstances Resuscitation 2005 8 135 170 10.1016/j.resuscitation.2005.05.003 16321711

[B5] Safar P Bircer N Cardiopulmonary cerebral resuscitation: basic and advanced cardiac and trauma life support: an introduction to resuscitation medicine 1988 3 London: W.B. Saunders

[B6] Arrich J Clinical application of mild therapeutic hypothermia after cardiac arrest Crit Care Med 2007 8 1041 1047 10.1097/01.CCM.0000259383.48324.35 17334257

[B7] Oddo M Ribordy V Feihl F Rossetti AO Schaller MD Chioléro R Liaudet L Early predictors of outcome in comatose survivors of ventricular fibrillation and non-ventricular fibrillation cardiac arrest treated with hypothermia: a prospective study Crit Care Med 2008 8 2296 2301 10.1097/CCM.0b013e3181802599 18664785

[B8] Cheung KW Green RS Magee KD State of the art- systematic review of randomized controlled trials of therapeutic hypothermia as a neuroprotectant in post cardiac arrest patients Can J Emerg Med 2006 8 329 333 10.1017/s1481803500013981 17338844

